# Clinicopathological factors of ovarian clear cell carcinoma: A single institutional analysis of 247 cases in China

**DOI:** 10.17305/bb.2024.10958

**Published:** 2024-08-13

**Authors:** You Wu, Xueyan Lyu, He Zhang, Miao Ao, Haixia Luo, Yanjia Chen, Yan Song, Bin Li

**Affiliations:** 1 National Cancer Center/National Clinical Research Center for Cancer/Cancer Hospital, Chinese Academy of Medical Sciences and Peking Union Medical College, Beijing, China

**Keywords:** Ovarian clear cell carcinoma (OCCC), recurrence, clinicopathological features, nomogram, immunotherapy

## Abstract

Ovarian clear cell carcinoma (OCCC) is a subtype of ovarian cancer with a poor prognosis that often shows resistance to chemotherapy. This study retrospectively analyzed 247 patients with OCCC who were admitted to the Cancer Hospital of the Chinese Academy of Medical Sciences (CAMS) between August 2007 and August 2023. Univariate and multivariate Cox regression analyses were used to identify clinicopathological factors associated with OCCC, and a nomogram prediction model was developed to predict OCCC patient survival outcomes. Kaplan–Meier survival analysis was used to compare survival outcomes among patients with recurrent disease. Compared with systemic therapy, secondary debulking surgery significantly improved the postrecurrence survival (PRS) rate (*P* ═ 0.006). Subgroup analysis revealed that the survival benefit was more pronounced in patients with recurrence and satisfactory tumor shrinkage (*P*_PRS_ ═ 0.01, *P*_PFS2_ ═ 0.047). The multivariate analysis revealed that positive preoperative ascites, incomplete remission following initial treatment, and undergoing more than six cycles of postoperative chemotherapy were independent prognostic factors affecting overall survival (OS) outcomes. Additionally, patients with a positive PD-L1 test who received immunotherapy did not experience relapse during the follow-up period. In conclusion, the secondary clearance procedure offers significant benefits for patients with recurrent OCCC, and patients may experience a survival benefit from supplemental immune or targeted therapy at the end of chemotherapy. The development of a personalized treatment plan can help achieve precise treatment, improve prognosis, and enhance patients’ quality of life.

## Introduction

Ovarian cancer (OC) has the lowest incidence among the three most common malignant tumors of the female reproductive system. However, OC demonstrates the highest rates of relapse and mortality [1]. Ninety percent of OCs originate from epithelial cells. According to Global Cancer Statistics 2024 (GLOBOCAN 2024), there are approximately 324,398 new cases of OC and 206,839 related deaths worldwide each year [2]. In China, approximately 55,000 new cases of epithelial OC (EOC) and 38,000 associated deaths were reported in 2023, both higher than the numbers reported in 2018 (53,000 new cases and 31,000 deaths) [3]. EOC exhibits different histological subtypes, including high-grade plasma carcinoma, clear cell carcinoma, endometrioid carcinoma, low-grade plasma carcinoma, and mucinous carcinoma. Ovarian clear cell carcinoma (OCCC) is a distinct pathological type of EOC that accounts for approximately 5%–25% of EOC cases [4]. There are significant racial and geographic disparities in the incidence of OCCC, with the lowest incidence in Black patients and the highest in Asian patients [5, 6]. OCCC has unique genetic features, such as frequent mutations in ARID1A and PIK3CA, MET amplification, and rare p53 mutations, distinguishing it from other EOC subtypes [7–9].

As a relatively chemoresistant subtype, OCCC is usually characterized by wild-type TP53 [10]. However, further clarification is required regarding whether chemotherapy resistance is associated with the mutational status of the TP53 gene [11]. Most patients with a better prognosis are in the early stages at OCCC diagnosis. Compared with other EOC subtypes, advanced OCCC has a worse prognosis because of a low response rate to chemotherapy [12]. In a previous study, the response rate to chemotherapy in patients with platinum-sensitive recurrent OCCC was less than 10%, and the objective response rate to chemotherapy in patients with platinum-resistant relapsed OCCC was only 1% [13]. Therefore, the treatment of OCCC presents significant challenges. In particular, there is a lack of effective treatments for patients who experience relapse.

In this study, we retrospectively analyzed the clinicopathological characteristics of 247 patients with pathologically confirmed OCCC who were treated at our center over 16 years to investigate their clinical features and the factors affecting their prognosis. We also compared the efficacy of different treatment regimens for patients with recurrent OCCC and preliminarily analyzed the effects of immunotherapy on patients with OCCC. Our study aimed to provide up-to-date information about the current treatment paradigm and propose hypotheses supporting rational treatment strategies.

## Materials and methods

### Patients

Data for this retrospective study were obtained from patients diagnosed with primary OCCC at the Cancer Hospital of the Chinese Academy of Medical Sciences (CAMS) between August 2007 and August 2023. The diagnosis of OCCC was determined based on the World Health Organization (WHO) criteria for determining histiocyte type and was confirmed by at least two pathologists. The inclusion criteria were as follows: pathologically confirmed clear cell carcinoma of the ovary; older than 18 years; radical surgery as the main treatment; good compliance; willingness to cooperate with related examinations; and close follow-up. The exclusion criteria were as follows: the presence of other primary malignancies; incomplete clinical and follow-up data; discontinuation of treatment according to guidelines or failure to complete a standard full course of therapy; the presence of serious comorbidities or other diseases that interfere with the assessment of survival and treatment efficacy; and pregnancy or lactation. Identifying patient information was kept confidential, per the Declaration of Helsinki. The study was approved by the Institutional Review Board, and the requirement for informed consent was waived because of the retrospective design.

The following information was collected from the medical records of eligible patients: age at initial treatment; preoperative routine blood test and biochemical examination results; pathological examination results of OCCC tissue, including lymph node status and degree of differentiation, as published by the American Joint Committee on Cancer (AJCC); and PD-L1 expression status, if the patient was tested. Additionally, surgical procedures performed, the presence of ascites, the residual tumor size, the presence of comorbid endometriosis, the adjuvant chemotherapy regimen, the duration of follow-up, recurrence, and survival were recorded. TP53 status was not addressed in this study.

Information regarding subsequent diagnosis and treatment plans, as well as subsequent survival and recurrence patterns, was collected for patients with recurrence. All tumors were staged according to the International Federation of Gynecology and Obstetrics (FIGO) staging system of 2014. Disease staging was retrospectively classified for patients treated before 2014 according to surgical and pathological assessments. Optimal debulking was defined as a residual tumor with a maximum diameter of ≤1 cm post-surgery.

### Follow-up

Patients underwent their first postoperative review one month after surgical resection, followed by recommendations for tumor marker testing, enhanced CT or other imaging examinations, medical history inquiries, and physical examinations every three months within the first two years post-surgery. Semiannual evaluations were conducted thereafter. The data mentioned above were retrospectively reviewed through the electronic medical records system, independently collected by two researchers, and integrated to obtain patients’ overall survival (OS), post-recurrence survival (PRS), and second progression-free survival (PFS-2) outcome data. OS was defined as the time from surgical staging or debulking surgery to death or the last follow-up date if the patient was still alive. PRS was defined as the time from the first relapse to disease progression. PFS-2 was defined as the time from the start of initial treatment to the second relapse. Relapse was defined as an imaging recurrence after a patient achieved complete response (CR) or partial response (PR) upon treatment. Disease progression was defined as imaging recurrence during or within three months of a patient’s treatment. Follow-up continued until 30 August 2023. Patients who did not return to our institution for follow-up were contacted by telephone by the researchers to confirm recurrence and survival information. Complete follow-up information was available for all enrolled patients.

### Statistical analysis

Statistical analyses were performed using R software (version 4.3.1; https://www.R-project.org). Comparisons between groups of continuous variables were made using one-way ANOVA or the Kruskal–Wallis H test, depending on the data distribution. Categorical variables were compared using the χ2 test or Fisher’s exact test. Univariate and multivariate Cox regression analyses were performed using patient OS as an outcome to identify risk factors for OCCC. A nomogram prediction model was subsequently developed. Factors included in the multivariate analyses were all factors with a *P* value of less than 0.1 in the univariate analyses and the relevant prognostic factors for OCCC mentioned in the guidelines and consensus. *P* values <0.05 were considered to indicate significance, and all reported *P* values were two-tailed.

## Results

### Patient information and clinical features

A total of 247 patients with OCCC were included in this study. The mean age at diagnosis was 51.97 ± 9.04 years (ranging from 29 to 76 years). Among them, 145 patients (58.7%) were in FIGO Stage I, 41 patients (16.6%) were in Stage II, 47 patients (19%) were in Stage III, and 14 patients (5.7%) were in Stage IV. This finding indicates the predominance of early-stage disease. The tumor size was greater than 10 cm in 97 patients (39.3%) and less than 5 cm in 70 patients (28.3%). Sixteen (6.47%) patients had concurrent endometriosis according to the criteria set by Sampson and Scott [5]. The median follow-up time for all surviving patients was 67 months (ranging from 5 to 196 months).

Prior to surgery, 28 patients (11.3%) received neoadjuvant therapy. All patients underwent surgical treatment, with 12 individuals (4.9%) undergoing laparoscopic surgery and 235 (95.1%) undergoing open surgery. Lymph node dissection was not performed in 56 patients (22.7%) because of their advanced disease stage. However, lymph nodes that were enlarged or larger than 1 cm to the naked eye were removed intraoperatively in conjunction with imaging and intraoperative exploration. At this point, R0 was achieved with no residual sarcomeric lesions in the abdominal cavity. Optimal surgical cytoreduction was achieved in 229 patients (92.7%), whereas 18 patients (7.3%) had suboptimal debulking results. Lymph node metastasis occurred in 24 patients (9.7%), and 79 patients (32%) had poorly differentiated tumors.

All patients received postoperative adjuvant chemotherapy, with 34 individuals (13.8%) receiving more than six cycles of adjuvant chemotherapy (Table 1). Moreover, four patients with advanced-stage disease who tested positive for PD-L1 expression received immune maintenance therapy after chemotherapy for 6–24 months; there has been no recurrence since follow-up (10–38 months) in these four patients.

**Table 1:** Baseline characteristics of 247 OCCC patients

**Characteristics**	**FIGO stage**				***P* value**
***n***	**I**	**II**	**III**	**IV**	
	**145**	**41**	**47**	**14**	
Age, mean ± SD	51.19 ± 8.95	51.76 ± 8.77	53.26 ± 9.28	56.36 ± 9.20	0.148
Endometriosis, *n* (%)					0.571
No	135 (54.7%)	39 (15.8%)	45 (18.2%)	12 (4.9%)	
Yes	10 (4%)	2 (0.8%)	2 (0.8%)	2 (0.8%)	
Ascites, *n* (%)					<0.001
No	139 (56.3%)	39 (15.8%)	32 (13%)	12 (4.9%)	
Yes	6 (2.4%)	2 (0.8%)	15 (6.1%)	2 (0.8%)	
Hydrothorax, *n* (%)					0.031
No	144 (58.3%)	40 (16.2%)	43 (17.4%)	13 (5.3%)	
Yes	1 (0.4%)	1 (0.4%)	4 (1.6%)	1 (0.4%)	
CA125, *n* (%)					<0.001
>35	80 (32.4%)	29 (11.7%)	40 (16.2%)	12 (4.9%)	
≤35	65 (26.3%)	12 (4.9%)	7 (2.8%)	2 (0.8%)	
Maximum diameter of the mass, *n* (%)					0.438
≥ 10 cm	52 (21.1%)	15 (6.1%)	24 (9.7%)	6 (2.4%)	
5–10 cm	47 (19%)	16 (6.5%)	14 (5.7%)	3 (1.2%)	
≤ 5 cm	46 (18.6%)	10 (4%)	9 (3.6%)	5 (2%)	
Surgical approach, *n* (%)					0.246
Laparotomy	135 (54.7%)	41 (16.6%)	45 (18.2%)	14 (5.7%)	
Laparoscopy	10 (4%)	0 (0%)	2 (0.8%)	0 (0%)	
Satisfactory tumor reduction at first operation, *n* (%)					<0.001
Satisfactory	143 (57.9%)	39 (15.8%)	35 (14.2%)	12 (4.9%)	
Unsatisfactory	2 (0.8%)	2 (0.8%)	12 (4.9%)	2 (0.8%)	
Effect, *n* (%)					<0.001
CR	143 (57.9%)	40 (16.2%)	35 (14.2%)	12 (4.9%)	
PR	2 (0.8%)	1 (0.4%)	12 (4.9%)	2 (0.8%)	
Lymph node metastasis, *n* (%)					<0.001
Unknown	29 (11.7%)	11 (4.5%)	15 (6.1%)	1 (0.4%)	
No	116 (47%)	26 (10.5%)	15 (6.1%)	10 (4%)	
Yes	0 (0%)	4 (1.6%)	17 (6.9%)	3 (1.2%)	
Chemotherapy cycles, *n* (%)					0.073
≤ 6 cycles	132 (53.4%)	32 (13%)	38 (15.4%)	11 (4.5%)	
> 6 cycles	13 (5.3%)	9 (3.6%)	9 (3.6%)	3 (1.2%)	

OCCC: Ovarian clear cell carcinoma; CR: Complete response; PR: Partial response; FIGO: International Federation of Gynecology and Obstetrics.

### Identification of prognostic factors and prediction modeling

**Figure 1.**
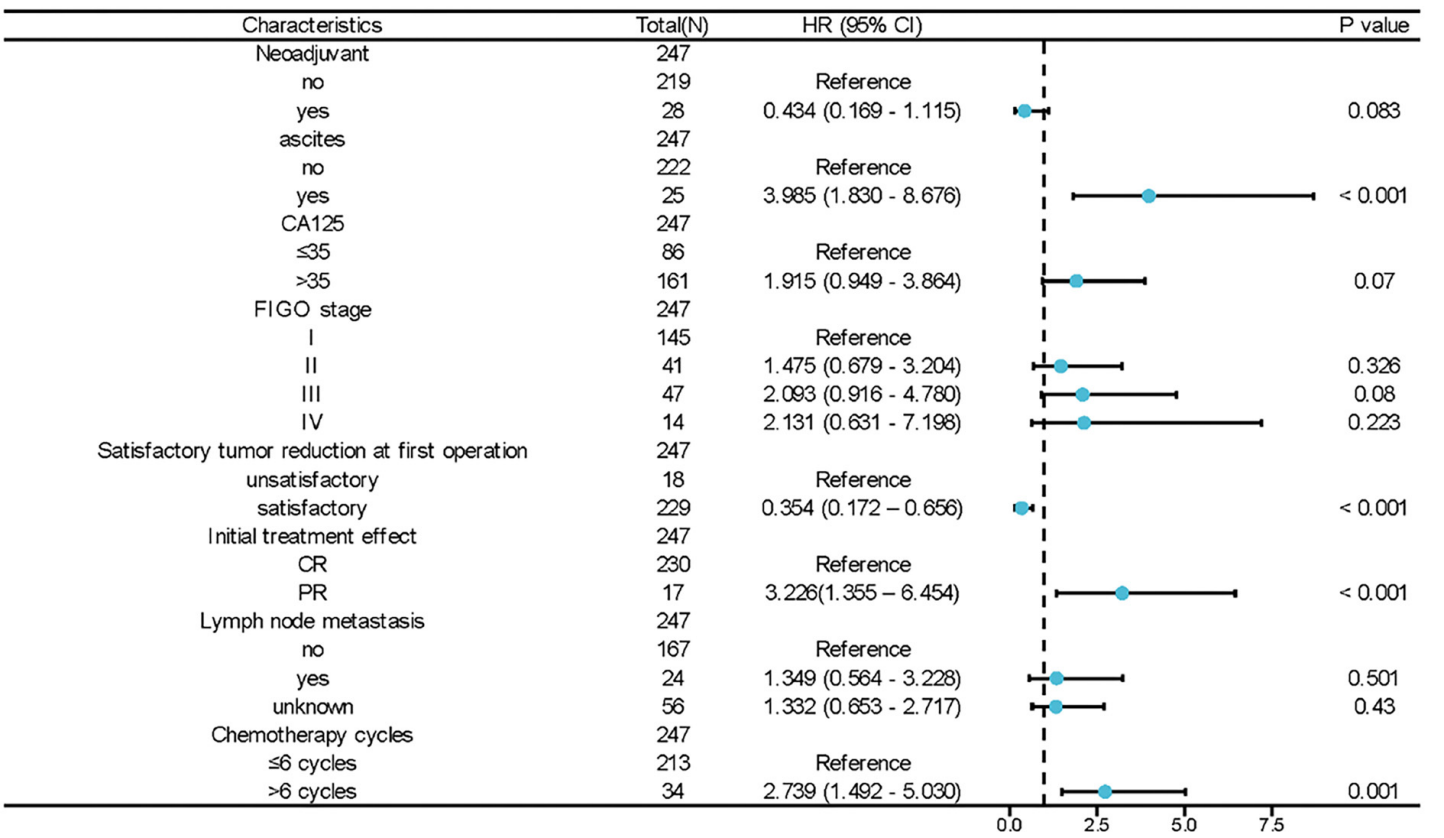
**Forest plot of multivariate Cox regression analysis of prognostic correlates of OCCC.** OCCC: Ovarian clear cell carcinoma; CR: Complete response; PR: Partial response; FIGO: International Federation of Gynecology and Obstetrics.

Five risk factors associated with poor prognosis were first identified in the univariate analysis. Eight factors demonstrated significance (*P* < 0.05) in the univariate analysis: neoadjuvant chemotherapy, ascites, CA125, FIGO staging, satisfactory tumor reduction at initial surgery, initial treatment efficacy, lymph node metastasis, and the number of chemotherapy cycles. Further multivariate Cox analysis revealed that positive preoperative ascites [HR ═ 3.985, 95% CI: 1.830–8.676, *P* < 0.001], non-CR to initial treatment [HR ═ 3.226, 95% CI: 1.355–6.454, *P* < 0.001], and postoperative adjuvant chemotherapy for more than 6 cycles [HR ═ 2.739, 95% CI: 1.492–5.030, *P* ═ 0.001] were independent risk factors for poor prognosis (Supplementary Information 1). The results of the multifactorial analyses were further demonstrated via forest plots (Figure 1).

**Figure 2.**
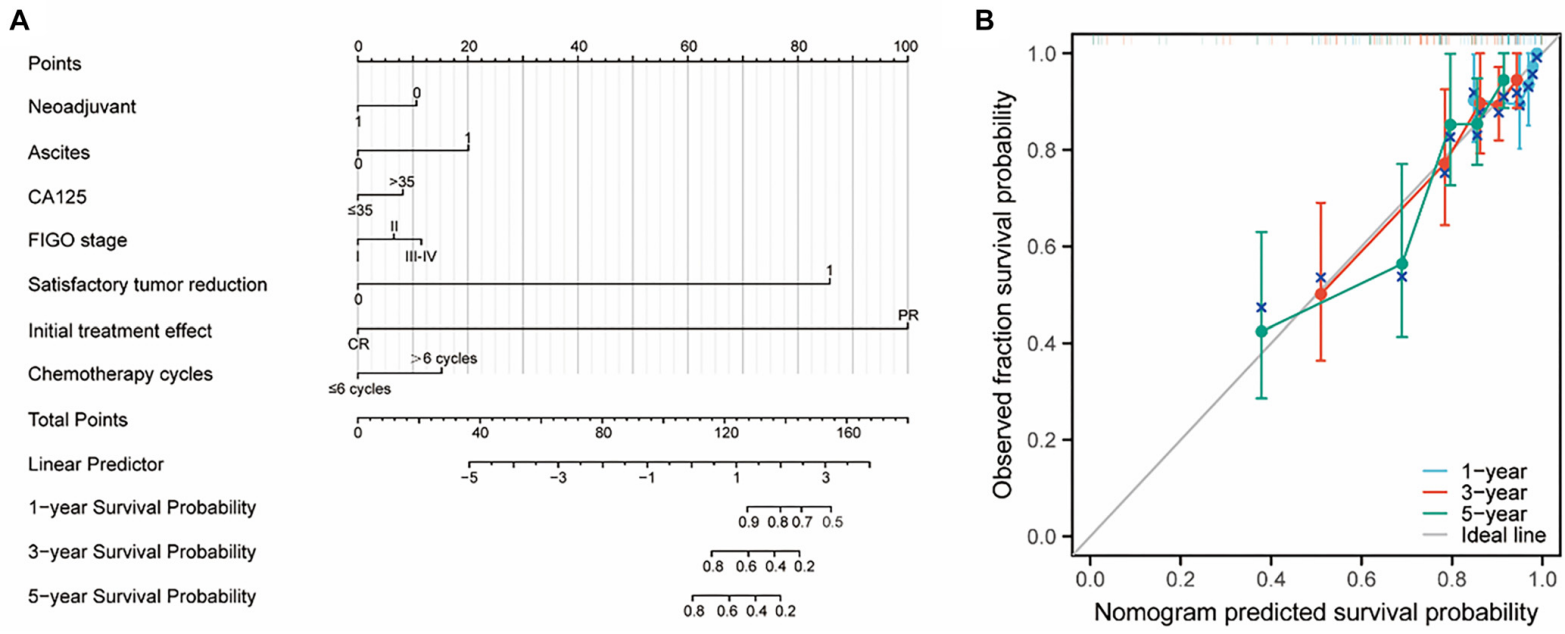
(A) A nomogram prediction model for factors associated with OCCC prognosis; (B) Prognostic calibration curves for the nomogram prediction model (1-, 3-, and 5-year survival rates). OCCC: Ovarian clear cell carcinoma; FIGO: International Federation of Gynecology and Obstetrics.

We incorporated the above prognostic factors into a nomogram prediction model, and the nomogram model (Figure 2A) and calibration curves (Figure 2B) showed good agreement. The risk proportions of each prognostic factor included in the model and the time-dependent receiver operating characteristic curve of the nomogram prediction model are shown in Supplementary Information 2, with areas under the curve of 0.717 for one year, 0.770 for three years, and 0.784 for five years. The calibration C-index was 0.725 after bootstrapping the model, indicating that the model has good predictive ability.

### Analysis of recurrence patterns in different treatment regimens

We subsequently conducted further subgroup analyses of the 74 patients who experienced recurrence. We divided patients into four groups according to the different treatment regimens adopted after relapse (Table 2): chemotherapy alone, chemotherapy plus surgery, chemotherapy plus immunotherapy, and chemotherapy plus bevacizumab groups. All patients who fully underwent secondary cytoreduction experienced a single recurrence, and 12 (44.4%) demonstrated satisfactory secondary cytoreduction results. Moreover, five patients underwent two or three procedures for recurrent disease.

**Table 2:** Information on OCCC patients with recurrence (*n* ═ 74)

**Treatments**	**Chemotherapy**	**Chemotherapy + surgery**	**Chemotherapy + immunotherapy**	**Chemotherapy + bevacizumab**
*n*	32	27	9	6
Age, median (range)	53.5 (38–70)	50 (34–64)	57 (46–64)	47.5 (34–61)
*FIGO stage at diagnosis (%)*				
Early (I+II)	23 (71.9%)	20 (74.1%)	2 (22.2%)	1 (16.7%)
Advanced (III+IV)	9 (28.1%)	7 (25.9%)	7 (77.8%)	5 (83.3%)
Platinum resistant recurrence (%)	4 (12.5%)	5 (18.5%)	6 (66.7%)	1 (16.7%)
*Number of recurrent tumor lesions*				
Single	13 (40.6%)	27 (100%)	4 (44.4%)	2 (33.3%)
Multiple	19 (59.4%)	0 (0%)	5 (55.6%)	4 (66.7%)
*Recurrence pattern*				
Within pelvis (%)	10 (31.2%)	17 (63%)	4 (44.4%)	0 (0%)
Out of pelvis (%)	22 (68.8%)	10 (37%)	5 (55.6%)	6 (100%)
Complete secondary cytoreductions (%)		12 (44.4%)		
PD-L1, CPS			2(1–10)	
ORR	25%	74.07%	33.3%	66.7%
Follow-up time (months), median (range)	99.5 (13–196)	85 (24–182)	63 (25–124)	53.5 (25–73)
*Disease status at last follow-up*				
Dead (%)	23 (71.9%)	7 (25.9%)	3 (33.3%)	2 (33.3%)
Alive with disease (%)	5 (15.6%)	5 (18.5)	5 (55.6%)	1 (16.7%)
No evidence of disease (%)	4 (12.5%)	15 (55.6%)	1 (11.1%)	3 (50%)
PFS2	8.5 (2–121)	14.5 (2–130)	12 (3–25)	13 (4–55)
Post-recurrence survival periods	22 (6–105)	3 (11–142)	45.5 (24–66)	25.5 (10–57)

OCCC: Ovarian clear cell carcinoma; FIGO: International Federation of Gynecology and Obstetrics.

Among the patients who received chemotherapy combined with bevacizumab, 66.7% (4/6 patients) had solid organ metastases, including liver (two patients, 33.3%) and lung (two patients, 33.3%) metastases; the remaining metastases were in the abdominal wall (one patient) and lymph nodes (one patient). The ORR was much higher than that of the patients who received chemotherapy alone.

In addition, chemotherapy and immunotherapy improved the treatment efficacy in patients who experienced platinum-resistant relapse, with an ORR of 33.3%.

### Treatment for patients with recurrence

**Figure 3.**
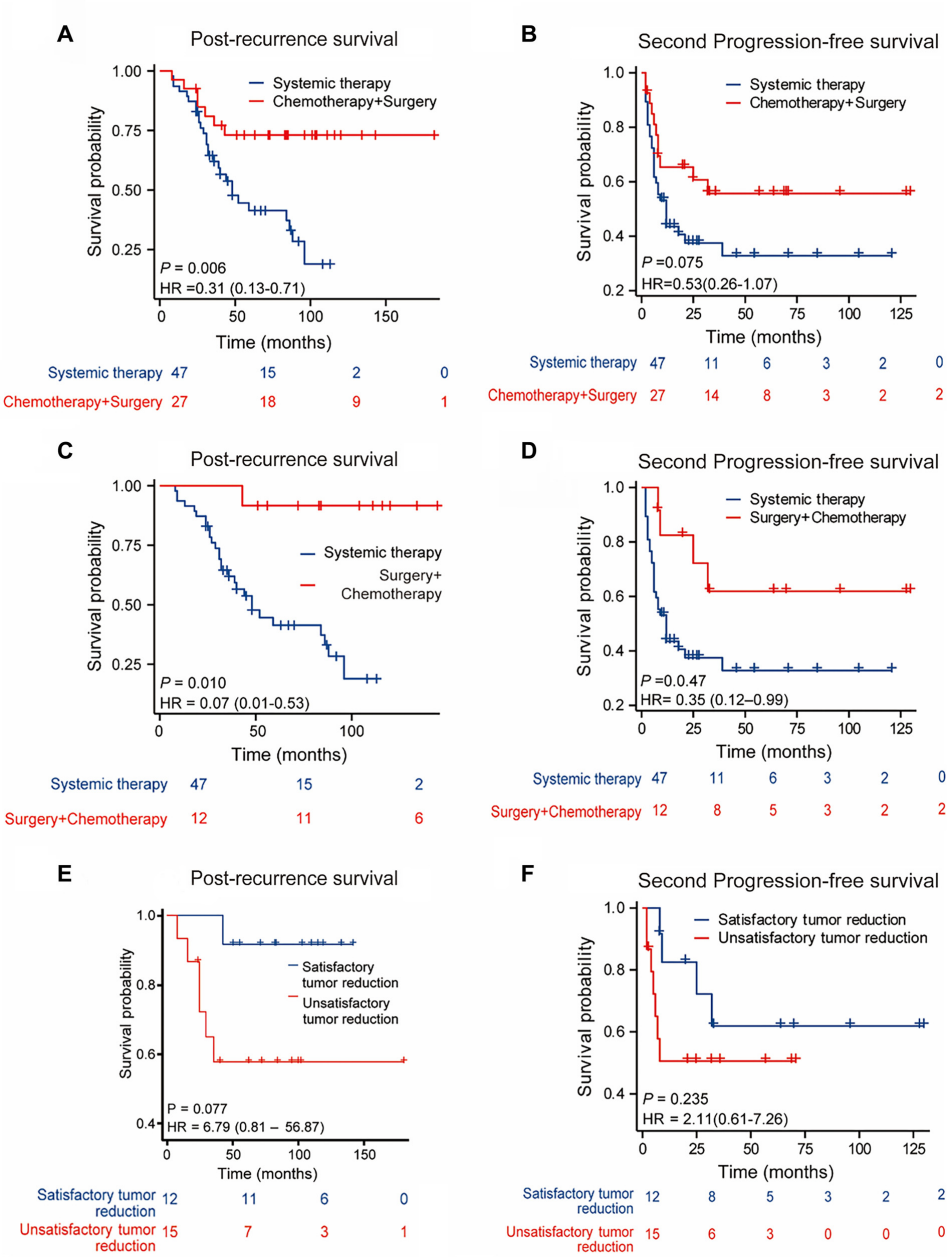
**Representative Kaplan–Meier survival curves.** (A) PRS was statistically superior in the surgery plus chemotherapy group compared to the systemic treatment group; (B) There was no significant difference in second disease-free survival between the two groups; (C and D) Patients who achieved complete resection at the time of the second clearance had longer post-recovery and second disease-free survival durations than those in the systemic treatment group; (E and F) Stratification according to the completion of the surgery: Complete resection group (without residual macroscopic tumor) vs incomplete resection group (with residual macroscopic tumor). The complete resection group had residual macroscopic tumors. Survival after recurrence and disease-free survival outcomes were worse in the incompletely resected group than in the wholly resected group, although the difference was not significant. PRS: Post-recurrence survival.

To further assess whether secondary tumor cytoreductive surgery (SCR) can affect the oncological prognosis of patients with recurrent OCCC, the patients were divided into the systemic treatment group (chemotherapy ± immunotherapy ± bevacizumab) and the chemotherapy plus surgery group. The two groups did not demonstrate significant differences in stage, initial surgery, or recurrence site distribution. Survival analysis revealed that the chemotherapy plus surgery group had better PRS than the systemic therapy group (*P* ═ 0.006, median 38 months vs 26.5 months) (Figure 3A). However, PFS2 did not differ between the two groups (*P* ═ 0.075, median 14.5 months vs 12 months) (Figure 3B).

Subgroup analyses were performed on patients who underwent satisfactory tumor cytoreduction. Relapsed patients who underwent satisfactory tumor cytoreduction had a greater PRS survival benefit than those who received systemic therapy (mPRS 44 m vs 26.5 m, *P* ═ 0.01) (Figure 3C). mPFS2 also improved (mPFS2 21 m vs 12 m, *P* ═ 0.047) (Figure 3D). Satisfactory tumor cytoreduction also resulted in greater PRS (mPRS 44 m vs 36 m, *P* ═ 0.077) (Figure 3E) and PFS2 (mPFS2 21 m vs 9 m, *P* ═ 0.235) (Figure 3F) survival benefits compared to those observed in patients with unsatisfactory tumor cytoreduction.

## Discussion

OCCC has long posed significant challenges for clinical diagnosis and treatment because of its unique biological characteristics and poor prognosis, especially in patients with recurrence [14]. Therefore, clinical studies aim to identify effective therapeutic strategies to address these challenges. In this study, we present a novel nomogram prediction model based on clinical departure that analyzes and incorporates seven predictors associated with poor prognosis in OCCC patients. Clinicians can score each patient’s indicators in combination with surgical pathological staging and auxiliary examinations and incorporate them into this model to evaluate patient prognosis. This approach will enable clinicians to provide more personalized guidance, especially during follow-up. The nomogram prediction model proposed in this study was shown to have a good predictive effect.

The age of onset for OCCC is younger than that for high-grade serous ovarian carcinoma, and OCCC is primarily diagnosed during the early stage [4]. In our study, the median onset age was 42 ± 3.5 years, with 75.3% of patients having stage I/II disease. Moreover, OCCC patients usually have concurrent endometriosis, with common pelvic adhesions and a high likelihood of tumor rupture during surgery. However, this study revealed that positive preoperative ascites cytology, but not the occurrence of intraoperative tumor rupture, affects patient prognosis.

OCCC also typically responds poorly to neoadjuvant chemotherapy [15, 16]. In our study, 11.3% (28/247) of patients, primarily those with stage III/IV OCCC, received neoadjuvant chemotherapy. Chemotherapy followed by surgery yielded satisfactory tumor reduction in 60% of patients. However, neoadjuvant chemotherapy did not affect patient prognosis. Satisfactory tumor reduction and achievement of complete remission were independent prognostic factors. The surgery scope was consistent with previous studies [17–20], including pelvic and abdominal para-aortic lymph node dissection. There was no significant difference in OS rates between patients with or without lymph node metastasis. Achieving R0 resection is the key to treatment. Interestingly, our study is the first to reveal that more than six initial chemotherapy cycles affect OCCC prognosis, which could be associated with tumor drug resistance. In this study, supplementation with immunotherapy or targeted therapy after chemotherapy improved the prognosis of this group of patients.

Given the rare incidence of OCCC and its relative resistance to chemotherapy, there is a lack of effective treatment options for patients who experience relapse [21–23]. Previous studies have confirmed that secondary debulking surgery to achieve R0 resection significantly prolongs the median PFS of patients with platinum-sensitive relapsed OC compared with chemotherapy alone [24–28]. In this study, 27 patients with relapse underwent secondary debulking surgery. A total of 81.5% of these patients had platinum-sensitive recurrent OCCC. Compared with systemic therapy, secondary debulking surgery significantly improved the median PFS2 and PRS outcomes, especially for those who achieved R0 resection. Notably, five of the patients with relapse underwent 2–4 secondary surgeries. The leading site of relapse for these patients was the pelvis, consistent with previous studies [29–31].

Nonetheless, secondary debulking surgery is not suitable for all patients who experience relapse, and most patients receive systemic therapy, especially those with extensive metastases and multiple relapses, for which R0 resection cannot be achieved. Numerous retrospective studies have confirmed that relapsed OCCC patients demonstrate poor sensitivity to chemotherapy, with ORRs ranging from 10% to 30% for various combinations of chemotherapy regimens [32, 33]. In this study, platinum-sensitive patients who experienced relapse had a greater rate of tumor control with chemotherapy. However, the response rate to altered second-, third-, and later-line chemotherapy regimens decreased as chemoresistance developed. Chemotherapy combined with bevacizumab or immunotherapy in PD-L1-positive patients (CPS > 1) improved the outcomes of patients with drug-resistant OCCC to some extent by prolonging their median PFS duration and improving their response rate. These findings suggest that chemotherapy combined with immunotherapy is an effective regimen for OCCC; however, the selection of suitable patients is necessary. There is also a need to explore additional immune response markers to determine treatment regimens in clinical practice.

Moreover, the management of OCCC becomes highly challenging upon relapse. The benefit of using PARP inhibitors for maintenance therapy in advanced OCCC remains controversial [34, 35] due to the small number of patients studied and the low frequency of BRCA mutations. In this study, four patients with PD-L1-positive advanced OCCC achieved CR after initial chemotherapy, followed by maintenance immunotherapy for 6–24 months. No recurrence was observed during the follow-up. This finding suggests that maintenance immunotherapy can mitigate the challenges of OCCC treatment; however, extensive prospective studies are warranted to confirm the role of maintenance immunotherapy in patients with OCCC.

### Strengths and weaknesses

In this study, we retrospectively analyzed the clinicopathological characteristics of a large cohort of 247 patients with OCCC at our center. For the first time, this study comprehensively analyzed the prognostic characteristics of OCCC patients treated with secondary debulking surgery and different systemic therapies, including immunotherapy and bevacizumab. Additionally, a novel nomogram prediction model was developed that can help clinicians make clinical decisions.

However, there are still some areas where this study could be improved. First, this study was a single-center retrospective study with several limitations. In particular, the number of patients who received a combination of immunotherapy and bevacizumab was small. Second, some confounding factors may have affected the accuracy of the results to some extent, such as the use of telephone follow-up for out-of-town patients, the inclusion of different immunotherapy and targeted therapy regimens in the same subgroup, and the lack of further subgroup analyses. Future large-scale multicenter prospective studies are still needed to confirm the relevant conclusions of this study.

## Conclusion

In conclusion, in patients with OCCC, positive ascites cytology, residual lesions after initial treatment, and the number of chemotherapy cycles are independent risk factors affecting prognosis. In response to different clinical treatment options, secondary clearance surgery, especially R0 resection, significantly improves the prognosis of patients with recurrence. Additionally, maintenance immunotherapy could be considered for patients with PD-L1-positive OCCC or as part of an individualized treatment strategy; however, further confirmation in extensive prospective studies is needed.

## Supplemental data

Supplementary data is available at the following link: https://www.bjbms.org/ojs/index.php/bjbms/article/view/10958/3421

## Data Availability

The datasets used and/or analyzed during the current study are available from the corresponding author upon reasonable request.
